# Investigating the Role of the Environment on Physical Activity Interventions (the InSPACE Project): Protocol for a Pooled Secondary Analysis of Randomized Controlled Trials

**DOI:** 10.2196/83151

**Published:** 2026-01-22

**Authors:** Amy J Youngbloom, Maya Rowland, Stephen J Mooney, Kaitlin Singer, Adam Szpiro, Brian E Saelens

**Affiliations:** 1 Department of Epidemiology, School of Public Health, University of Washington, 3980 15th Ave NE, Seattle, WA, 98195, United States, 1 2065431065; 2 Center for Child Health, Behavior and Development, Seattle Children's Research Institute, Seattle, WA, United States; 3 Department of Biostatistics, School of Public Health, University of Washington, Seattle, WA, United States; 4 Department of Pediatrics, School of Medicine, University of Washington, Seattle, WA, United States

**Keywords:** physical activity intervention, built environment, pooled analysis, physical activity, interventions, social environment, accelerometry

## Abstract

**Background:**

Physical activity (PA) interventions can increase levels of PA to help participants meet recommended levels. The impact of PA interventions may be affected by an individual’s neighborhood environment, including attributes such as walkability, crime rates, or greenspace availability, but research to date has lacked the power and geographic spread to adequately assess the role of the environment.

**Objective:**

The Interventions Supporting Physical Activity and the Environment (InSPACE) study used the Automatic Context Measurement Tool (ACMT) to gather environmental measures for participants around their home address in completed lifestyle intervention trials across the United States, then pooled and harmonized demographic and device-based activity data, creating a dataset for use in assessing the moderation effect of neighborhood attributes on interventions to increase PA.

**Methods:**

PA intervention trials were recruited from across the United States, and trialists were instructed in the use of the ACMT to geocode and collect prespecified environmental measures. The InSPACE research team gathered deidentified data from trialists, including demographics, raw accelerometry data, and ACMT-generated environmental measures and harmonized data to create a pooled dataset of PA intervention trial participants.

**Results:**

As of August 2025, a total of 39 PA intervention trials have been recruited and data from 31 of these trials have been processed and harmonized, creating a current pooled dataset of 4471 participants with any harmonized data, of whom 4360 (97.5%) have linked environmental data and 2208 (49.4%) have specified (3 days of at least 8 hours per day) accelerometry data for both baseline and postintervention. Results from the primary analysis for the InSPACE project are expected to be published in late 2026.

**Conclusion:**

InSPACE will contribute to understanding the role of the environment in moderating the effect of interventions to increase PA. The protocols and processes of InSPACE can inform future projects in pooled data harmonization and analysis.

## Introduction

### Background

Physical activity (PA) is linked to metabolic health, diabetes, cardiovascular disease, and some types of cancer [1-4]. Getting adequate daily PA may also improve cognitive function and bone strength, reduce depression, and improve overall well-being [2,5,6]. Most adults and youth in the United States do not meet the recommended guidelines for PA [7,8].

PA interventions have the potential to increase PA in youth and adults. Such interventions can include goal setting, supervised instructor-led exercise, educational sessions and information sharing, or peer support [9-17]. Meta-analyses have shown a positive mean effect of PA interventions, but the effect size varies significantly between types of interventions and population attributes [18-20]. Researchers have examined some potential modifiers of PA intervention effectiveness, including participant demographics and intervention type, but there has yet to be an extensive examination of environmental attributes as a moderator of PA intervention effectiveness, in part due to the lack of adequate data for such a study.

An individual’s neighborhood environment may affect their level of PA [21-23]. For example, a more walkable area around one’s home, as measured by land use, street connectivity and density, and greater access to sidewalks around one’s home is associated with more time spent in PA, specifically walking [24,25]. Living near a park or other urban green space can also promote more PA, especially for higher-income residents [26-28]. Physical disorder, such as vacant homes, litter, or vandalism, may reduce PA levels among residents [29]. Despite existing research on neighborhood environment and PA, and the growing prevalence of ecological models of behavior change that consider multiple levels of influence, there remains a gap in research examining how the neighborhood environment may influence the effectiveness of PA intervention outcomes.

A few studies have considered the moderating role of neighborhood attributes on PA intervention effectiveness, but these have been limited to perceptions of walkability and the National Walkability score and have shown mixed results [30-32]. These studies were significantly limited by small sample sizes, limited access to objective neighborhood attribute measures, and lack of geographic and demographic heterogeneity. The lack of substantial research in this area emphasizes the need for a large, diverse pooled dataset of PA intervention trials with standardized environmental measures that can be more rigorously examined for their potential modifying effect. The goal of the Interventions Supporting Physical Activity and the Environment (InSPACE) project was to help fill this gap and create opportunities for research on the modifying effect of the built environment on PA intervention effectiveness.

### Objective

The InSPACE study seeks to assess the modifying effect of the neighborhood environment on the impact of interventions in increasing PA by pooling and analyzing data from completed PA intervention trials of varied geographies, contexts, and populations. The InSPACE team provided and instructed trialists to use the Automatic Context Measurement Tool (ACMT) to geocode participant address data and generate environmental data specific to the neighborhood around the home address of pooled trial participants [33]. This paper describes the protocols used to gather and harmonize demographic and accelerometry data across trials in the InSPACE study and the use of the ACMT to estimate residential environment measures for participants in InSPACE trials. These protocols may not only guide future research on environmental effect modification of PA intervention effectiveness, but also more broadly inform future pooling studies examining environmental, individual demographic, and device-based measurement of PA.

## Methods

### Scientific Advisory Council

The InSPACE study team is advised by a scientific advisory council (SAC) comprised of 7 PA intervention research experts who provided ongoing feedback on trial inclusion criteria, trial recruitment strategies, and data harmonization processes. SAC members also shared expertise regarding intervention research (in PA as well as other health behaviors) in diverse populations, including across income, race and ethnicity, urban and rural geography, and among youth and older adults. Finally, SAC members assisted with recruitment by reaching out to potential trialists in their professional networks and informed individual-level demographic variable harmonization.

### Inclusion Criteria

Initial trial inclusion criteria required trials to have at least 50 free-living participants 6 years of age and older, and have targeted increasing PA as part of an intervention. For data collection and harmonization purposes, trials must have collected PA data using accelerometers or pedometers, retained individual participant address data, linked participant demographic and PA data, and have participants in the United States. We included only trials conducted fully or mostly between 2010 and 2020 to align better with the timing of the available environment data and to avoid potential impacts from the COVID-19 pandemic. The intervention must have had an anticipated effect on PA, although PA did not necessarily have to be the primary or only target of change. For example, if a trialist hypothesized that an increase in PA would improve mental health and therefore developed a trial intervention that aimed to increase participants’ PA to improve their mental health, this trial would be eligible for InSPACE, provided all other inclusion criteria were met. Trials must have also had a control condition, expected at the time of trial design to be less effective in promoting PA than the intervention. The InSPACE investigator team examined the plausibility that the intervention could be affected by the neighborhood environment before confirming the trial eligibility for inclusion. All trials must have collected PA data for at least two time points: (1) at least once prior to the intervention and (2) at least once after the intervention was complete (postintervention), such that any activity occurring as part of the intervention (eg, an exercise class facilitated by the research team to which participants were encouraged to attend) was not included in either activity collection. Initial criteria excluded trials with any PA intervention facilitated by the intervention team or that took place at a prescribed location (eg, in a fitness center).

After the initial trial recruitment and in consultation with the SAC, eligibility criteria were expanded to include interventions with intervention team–facilitated PA interventions at prescribed locations, to reflect an ecological theory of behavior change that any PA may increase other self-initiated community-based PA as well, particularly if the community environment supports PA [34]. Additionally, criteria were expanded to include trials with control groups that were not inert but were different from the intervention in that the trialists hypothesized there would be differences in PA between intervention and controls at postintervention. Ultimately, if the trialist hypothesized that the intervention group would have a greater increase in PA than the control group, the trial was eligible regardless of the amount of intervention team–facilitated PA within the intervention, or the content or intensity of the control group. With the inclusion of intervention-team or instructor-led PA trials, it was important to ensure that postintervention accelerometry data did not overlap with any intervention-team or instructor-led PA.

Our final trial inclusion change, adopted to broaden our trial population after an initial trial recruitment pass exhausted our trial list, was to lower the minimum number of participants per trial from 50 to 30. The full list of the final inclusion and exclusion criteria, as well as changes that were made in inclusion or exclusion criteria, is listed in Table 1. As the inclusion criteria evolved, literature was rereviewed and investigator teams from newly eligible trials were contacted.

**Table 1.** Interventions Supporting Physical Activity and the Environment trial inclusion and exclusion criteria.

Inclusion criteria category	Final inclusion and exclusion criteria details	Changes to inclusion and exclusion criteria
Population	Inclusion: Free-living humans in the contiguous United States with an available geocodable home address who are 6 years of age or older. Exclusion: Animal trials or populations that reside outside of the contiguous United States.	Original criteria did not specify the contiguous United States. This was changed as we realized some of our national measures do not include data for AK^a^ and HI^b^.
Sample size	Inclusion: Trial must have enrolled 30 or more participants (or 30 or more dyads). Exclusion: Trials with fewer than 30 participants (or fewer than 30 dyads) enrolled.	Original criteria only included trials with 50 or more participants (or 50 or more dyads). After exhausting the list of trials with 50 or more, we adjusted our criteria to include smaller trials.
Interventions	Inclusion: (1) PA^c^ is targeted for increases, regardless of whether PA is one of the primary aims of the trial. (2) Focus is on manipulating PA through variation in intervention delivery, content, and other factors, including those that target other issues or health outcomes and include PA. (3) Interventions can include some intervention-team or instructor-led PA that participants are able to, or are expected to attend. Exclusion: Intervention is very specific to single systems or parts of the body.	Trials that included any investigative team or instructor-led PA were not originally included and were added at the encouragement of our SAC^d^.
Trial design	Inclusion: (1) There should be an expectation of superiority for the intervention arm and NOT just noninferiority. (2) There is an expectation that the delta in PA between study arms could be influenced by the neighborhood environment. (3) Trial must be a randomized controlled trial. (4) Some control arm that is not only a different dose of prescribed physical activity.	Original criteria limited to inert control groups (waitlists, no-contact, etc).
Physical activity measurement timing	Inclusion: Free-living PA both before and after the intervention, and in any follow-ups after the intervention. Okay if PA measurement overlaps with some intervention time.^e^ Inclusion: (1) PA outcome is measured outside of intervention team–facilitated PA time both at baseline, post, and other follow-ups.^f^ (2) Measurement captures self-initiated activity that might be in the community.^f^ Exclusion: PA is measured only within specific intervention settings.^f^	Trials with instructor-led PA were not initially included. They were later included at the encouragement of our SAC only if the timing of their PA measurement met criteria.
Data collected and available	Inclusion: (1) Device measured PA- Accelerometer measured PA OR Pedometer measured PA or steps if collected data per minute (eg, not by week or day) AND in adults 18 years or older. (2) Address data. (3) Minimum demographic data (age, race, and sex). Exclusion: Self-report PA only. (2) Trials using pedometer measurement in those younger than 18 years or pedometer trials with steps aggregated by day or larger unit. Data are no longer available.	Initially excluded trials that assessed PA via pedometer.
Trial dates	Inclusion: Intervention began in 2010 or later and mostly completed pre-COVID-19. Exclusion: (1) Intervention began prior to 2010. (2) Participants were still receiving the intervention during COVID-19. Follow-ups after March 2020 allowed.	Did not change.

a AK: Alaska.

b HI: Hawaii.

c PA: physical activity.

d SAC: scientific advisory council.

e Outcomes for trials without an instructor-led component.

f Outcomes for trials with an instructor-led component.

### Trial Recruitment

The InSPACE research team used the National Institutes of Health (NIH) eReporter and ClinicalTrials.gov websites, as well as word-of-mouth, to identify potentially eligible intervention trials. ClinicalTrials.gov searches were conducted by Seattle Children’s librarians and reviewed by the InSPACE team. The principal investigators or leads of trials considered potentially eligible by the InSPACE team were contacted by the InSPACE principal investigator by email. The initial email included a one-page document describing the study and a brief introduction. If other members of the investigator team or SAC had previously collaborated with the trialist, these colleagues were CC’d on the introduction email and asked to “bump” the initial contact by responding and prompting the trialist to consider learning more about and engaging in InSPACE. If trialists responded with interest, basic eligibility questions were asked via email (eg, whether the participant’s home address and accelerometry data were still available). If still eligible, a conference call was organized to further describe InSPACE participation and further screen for eligibility and interest. Thereafter, trial information was brought back to the investigative team for review and to confirm eligibility. After confirmation, trialists were reapproached with a participation inclusion offer.

Offers included a contract to be established between the trialist’s institution and Seattle Children’s Research Institute. These contracts typically established a 6-month contract period and paid US $6000 in direct costs plus facilities and administrative costs for the first trial and US $2000 in direct costs for each additional trial for teams who contributed data from multiple trials. Contracts were milestone-based (eg, contract in place, Institutional Review Board [IRB] approval, ACMT training completed, and data transferred). Because of the unusual nature of these small subawards, the Seattle Children’s contracts team offered meetings with partner institutions early in the contracting process to answer questions ahead of time and prevent avoidable delays.

### Measures

#### Measures of Environmental Context

Participating trialists worked with the InSPACE team to generate residential environmental measures for each trial participant. This measurement collaboration leveraged the ACMT, an open-source tool for linking environmental data to any point in the United States (eg, participant addresses for InSPACE) that was provided to each trial team. The ACMT includes an RShiny-driven user-friendly interface that does not require prior geospatial software experience to link environmental measures. The ACMT’s geocoding and linkage occur locally such that trial teams can generate and share the measures without sharing personally identifiable participant data with the InSPACE team or a third-party geocoding site [35]. The ACMT is described more fully elsewhere [33].

To run the ACMT, a user must have administrative privileges on their computer. In some cases, our trialist partners were not administrators on their computers or encountered institutional firewalls that prevented them from installing and running the ACMT without IT assistance. Additionally, because geocoding and data linkage involve large geographic files, ACMT-driven linkage often requires considerable disk space and memory resources. While requirements depended on the number of state files that needed to be downloaded, in most cases, 100 GB of available disk space and 8 GB of memory were adequate for the ACMT to function properly on trialists’ computers. Despite challenges in working through firewalls and administrator privileges, none of the recruited trials dropped out of the InSPACE study due to technical issues with the ACMT.

Trialists who had not already used their own geocoding systems to identify participants’ home locations (this was rare) used the geocoder embedded in the ACMT to generate the latitude and longitude values for each participant’s address. Trialists who used the ACMT geocoder were asked to update addresses that received geocode ratings higher than 0 (the PostGIS geocoder embedded in the ACMT uses 0 to indicate the highest level of confidence in the accuracy of the geocode; Corti et al [36]). To improve the geocoding accuracy, trialists used the ACMT to edit and improve the address text, including removing apartment numbers, fixing obvious typos, and spelling out street names and cardinal directions. If these initial edits did not improve the geocode to a rating of 0, the ACMT mapping tool provided the user a way to view geocoded locations on an OpenStreet map (embedded within the ACMT so as not to reveal addresses to third parties) and to document a note as to whether the geocode appeared accurate (mapped to the correct street and city) or not. This geocode checking process helped improve the quality of the geocodes and deterred trialists from using web-based third-party mapping tools to verify addresses and geocodes.

Next, trialists used the ACMT to link environmental data from the datasets listed in the table below (Table 2) to the geocoded home addresses. For most datasets, only 1 year of data was available. Notable exceptions include American Community Survey (ACS) 5-year estimates, for which data are provided in 5-year increments, starting with 2005‐2009. The National Land Cover Database (NLCD) includes data every 2‐3 years from 2004 through 2019, regional price parity was available for all years between 2008 and 2018, and gentrification was available for 2000 (indicating gentrification from 1990 through 2000) and 2010 (indicating gentrification status from 2000 to 2010). For datasets with multiple years of data, we chose 2015 as the a priori estimated midpoint of enrollment years for all recruited trials and linked environmental data for the year of available data closest to 2015 (ie, default year for NLCD data was 2016—the closest to 2015 of the available years of data; for ACS this meant pulling the 5-year span of data with a midpoint of 2015 [2013‐2017]). In addition to the default year(s) of data, trialists were asked to pull environmental data that most closely aligned with the trial’s first year of enrollment. For example, if a trial enrolled participants from 2018to 2019, they would pull data for ACS for both the 5-year span of data with a midpoint of 2015 (ACS 5-year data for 2014‐2018; ie, the default year for environmental data), as well as the 5-year span whose midpoint most closely aligned with their enrollment (ACS 5-year data for 2016‐2020; ie, ACS data with a midpoint of 2018). The same trial would also pull NLCD data for both 2016 and 2019 (data for 2018 are not available for NLCD), CDC (Centers for Disease Control and Prevention) Places data for 2017 (closest available year to the default year of 2015) and 2018, and Regional Price Parity data for 2015 and 2018.

**Table 2.** Environmental measure datasets pulled for Interventions Supporting Physical Activity and the Environment trials.

Dataset	Description	Years available	Data summary method
American Community Survey [37]	Five-year pooled sociodemographic census data.	2009 through 2021 (default 2013‐2017)	Radial buffer interpolation (500 m, 1000 m, 5000 m)
Modified Retail Food Environment Index [38]	Census tract estimates of the ratio of healthy food outlets to total food outlets.	2011^a^	Radial buffer interpolation (500 m, 1000 m, 5000 m)
National Walkability Index [39]	Block group level ranking (1 to 20) of walkability, based on street intersection density, proximity to transit stops, and diversity of land use.	2019^a^	Radial buffer interpolation (500 m, 1000 m, 5000 m)
CDC Places [40]	Census tract-level estimates of population behaviors taken from survey responses to BRFSS.^b^	2017^a^, 2018	Radial buffer interpolation (500 m, 1000 m, 5000 m)
National Land Cover Database [41]	Satellite imagery is used to create pixel-by-pixel maps of land cover features, including forest, developed areas, open space, etc.	2004, 2006, 2008, 2011, 2013, 2016^a^, 2019	Radial buffer interpolation (500 m, 1000 m, 5000 m)
ParkServe [42]	Geographical information of parks throughout the United States. Used to calculate the distance to the nearest park (nonradial buffer measure), and the proportion of buffers that is park land.	2022	Radial buffer interpolation (500 m, 1000 m, 5000 m) for proportion of park land measure
CrimeRisk [43]	From Applied Geographic Systems, block group level risk ratio of various types of crime.	2022	Radial buffer interpolation (500 m, 1000 m, 5000 m)
Sidewalk and Crosswalk Availability [44]	National sidewalk and crosswalk presence dataset, created by applying machine learning to Google Street View images to create census tract-level estimates of the prevalence of sidewalks.	Latest imagery as of 2017	Radial buffer interpolation (500 m, 1000 m, 5000 m)
Regional Price Parity [45]	Price indices measuring geographic differences in the cost of housing, services, and more for a given metropolitan or state-wide area.	2008‐2018 (default year: 2015^a^)	Linked via MSA^c^ GEOID^d^ or state GEOID if participant resided outside of an MSA.
Gentrification [46]	Developed by the Urban Health Collaborative at Drexel University, this dataset categorizes each census tract as ineligible to gentrify, not gentrified, gentrified, intensely gentrified.	2000^a^, 2010^a^	Linked via census tract

a Default year, pulled by all trialists, regardless of enrollment year.

b BRFSS: Behavioral Risk Factor Surveillance System.

c MSA: metropolitan statistical area.

d GEOID: geographic identifier.

Due to the limited availability of many of the environmental datasets, there is not perfect temporal alignment between the year of participant enrollment and the year of environmental measures. We do not expect that this temporal misalignment will create significant bias, particularly given the evidence that environmental attributes, such as sidewalk availability, greenspace, and gentrification change very slowly [47-49]. Study metadata, including year of enrollment for participants, length of intervention, and year of follow-up, will also be available in the pooled data to allow for more precise temporal alignment so that future sensitivity analyses may assess potential bias due to temporal misalignment.

For most environmental data linkages, radial buffers were used to define a participant’s neighborhood, and environmental measures were computed using area-weighted averaging for the buffered area. For example, to calculate the walkability index for a buffer around a participant’s house that was comprised 50% of Block group A, 20% of Block group B, and 30% of Block group C, the ACMT calculates the weighted mean of the walkability score for Block groups A, B, and C using the proportion of the buffer that is comprised each Block group as that tract’s weight.

Across the lifespan, a substantial amount of physical activity occurs or originates near or around one’s home or neighborhood, with walking in particular most commonly occurring within 500 to 1000 m of the home [50,51]. Larger buffers may be necessary to more accurately capture neighborhood attributes in more suburban or rural areas, particularly for attributes such as green space and park access [52-54]. No buffer size fully captures the bandwidth at which neighborhood factors might influence PA; the most appropriate buffer to use may vary by age, urbanicity, and by the neighborhood environment factor [24,55-57]. Because all linkage was performed at one time, but future analyses might want to consider different buffer sizes, we selected 3 sizes for all area-buffered environmental data: 500, 1000, and 5000 m. Notable exceptions to the buffering approach include gentrification and Regional Price Parity (Table 2).

#### Demographic Harmonization

Trialists provided individual-level participant demographic data. While we anticipated that the available demographic variables would differ across trials, we requested that trials share baseline demographic data, including gender or sex, age, race, ethnicity, household size, or other variables that could help us determine household size, any measure or indicator of income and socioeconomic status, including income, educational level, insurance status, etc, and a variable to indicate which group the participant was randomized to. For trials where more than one household member was included (ie, parent-child dyads), we requested that a household ID be included.

A set of principles was developed to inform the harmonization of race and ethnic identity categories across trials. Race harmonization categories were based in part on review of trialists’ collection of race and ethnicity data and were further informed by racial harmonization methods used in prior research [58]. Race values were not assumed or assigned unless a participant was specifically asked and self-selected or affirmed in the race or ethnicity category. For example, in cases where a trial asked about race and ethnicity in a single question, participants’ ethnicity was marked as missing if they selected a race or ethnicity category other than Hispanic, and participants’ race was marked as missing if they selected only Hispanic. Participants who selected more than one race category were categorized as multiracial, and participants who selected “Other” for their race but did not specify any specific races or specified a race other than the designated categories were marked as Other for their race. Ultimately, the harmonized racial categories included: American Indian or Alaska Native, Asian, Black, Native Hawaiian or Other Pacific Islander, White, Multiracial, Other, or Missing (Table 3). Ethnicity categories were Hispanic or Latino or not Hispanic or Latino. In trials where a specific demographic population was recruited, and only individuals from that specific demographic group were eligible for the study, trialists typically did not record this information in their demographic dataset. For such cases, we imputed the relevant data (eg, for a trial whose inclusion criteria required participants to self-identify as Black women, we imputed their race as Black and their gender as female).

**Table 3.** Harmonized race values.

Harmonized race value	Raw race values
American Indian Alaska Native	Native American American Indian or Alaskan Native
Asian	South Asian Asian (having origins in any of the original peoples of the Far East, Southeast Asia, or the Indian subcontinent, including Cambodia, China, India, Japan, Korea, Malaysia, Pakistan, the Philippine Islands, Thailand, and Vietnam) Asian Indian Chinese Filipino Japanese Korean Vietnamese Other Asian
Black	African American Black
Native Hawaiian or Other Pacific Islander	Native Hawaiian or Other Pacific Islander (having origins in any of the original peoples of Hawaii, Guam, Samoa, or other Pacific Islands) Pacific or Hawaiian Native Hawaiian or Pacific Islander Guamanian or Chamorro Samoan Other Pacific Islander
White	Caucasian White Non-Hispanic White
Multiple	More than one race Multiple Multiple selected race categories Multiracial
Other	Other race or origin Other

Education harmonization was informed by the education categories used by trials in the pool. Education level for adults was combined and collapsed as needed to create six categories: (1) no schooling; (2) less than high school degree; (3) high school degree or General Educational Development; (4) some college, including technical school or associate’s degree; (5) bachelor’s degree; (6) some postgraduate education or more (Table 4). For trials that recruited children and collected data on parent education, we harmonized parent education according to these same 6 categories.

**Table 4.** Education harmonization mapping.

Harmonized education value	Raw education values from trial data
No schooling	No schooling
Less than high school degree	Less than 7th grade 8th grade or less Less than 8th grade Grades 0‐8 Grades 1‐8 Less than high school Some high school Junior high or middle school 8th grade or more, but less than high school Grades 9‐11 11th grade or less Partial high school
High school degree or GED	12‐13 years High school degree or GED^a^ Completed high school High school degree High school diploma or GED High school or equivalent (9-12) High school graduate High school
Some college, technical, or associate school	Some college 14‐15 years Trade or technical school Some college or vocational training 1‐3 years of college Associate’s degree Technical school certificate Some college or technical school 2 years of college, AA^b^ or technical school College (13-16) College or vocational or technical school 2-year college
Bachelor’s degree	College graduate 16‐17 years Completed college or university Bachelor’s degree College (BS^c^ or BA^d^) College degree College or university Graduated from college 4-year college
Some postgraduate education	Graduate degree 18 or more years Postgraduate training Completed graduate degree Postgraduate or professional degree Graduate work or higher Master’s degree Doctoral degree Postgraduate degree PhD or equivalent Postgraduate or advanced degree

a GED: General Educational Development.

b AA: Associate of Arts.

c BS: Bachelor of Science.

d BA: Bachelor of Arts.

Harmonizing participant-reported income data was challenging. Preharmonization tables provided no clear options for harmonizing income by collapsing or combining categories (as having categories was the common practice, such as <US $20,000, US $20,001-US $40,000, etc) due to vastly different ranges of income categories across trials. It was particularly problematic to determine how to harmonize across the highest income category option, given the variability across trials and the open-ended nature of the highest category (eg, >US $75,000 vs >US $300,000). Furthermore, because the purchasing power of income varies across regions, the same income level might represent substantially different economic resources for participants in trials in different geographic regions. Due to these complexities, we chose to use not to harmonize income at all for primary aims analyses, but rather to use a nonharmonized within-trial categorical income variable for confounding adjustment in analyses.

#### Physical Activity Harmonization

Each InSPACE trial and the associated intervention took place over its own distinct amount of time (eg, some trials lasted weeks and some months or years). Trialists provided individual-level accelerometry files for each data collection period. We defined baseline accelerometry data to include all accelerometry data collected prior to the start of the intervention. We defined the postintervention accelerometry data to be the first tranche of data collected after the end of the intervention period. Some trialists shared additional follow-up accelerometry data (eg, after noncontact follow-up periods), which we harmonized for use in future analyses. We processed the data using established definitions of valid accelerometry data, based on existing literature. Our primary definition of valid accelerometry data requires 8 hours of wear time per day for a given day to be valid, and 3 days of valid wear time for a given time period of data collection to be considered valid [59]. Baseline, postintervention, and any additional follow-up after postintervention accelerometry data were processed using R (R Core Team) as detailed below.

We requested accelerometry data in the rawest form available. For example, in most cases, Actigraph accelerometer (most common device used in pooled trials) files were shared in ActiLife’s native file format (agd), or as a csv file aggregated at no more than the 1-minute epoch level to ensure that the InSPACE team could implement consistent wear time and conversion to physical activity minutes processing protocols across all participants from different trials. Most (31/39, 79%) of trialists used hip-worn Actigraph accelerometers (models 7146, GT3X, or GT9X). However, data from wrist-worn Actigraph, thigh-worn activPAL3, and wrist-worn Actical and GENEactiv accelerometers were also included in the pooled accelerometry data (see below for differences in processing dependent on wear site).

While trials with only day-level accelerometry data were not considered eligible during screening, there were 2 trials that were recruited and entered into a contract believing that they had minute-level accelerometry data but ultimately were only able to locate day-level accelerometry data. In these cases, we confirmed with the trialists’ study team the specific wear time algorithm used as well as the activity count thresholds to determine whether these could rigorously be combined with the other harmonized and processed accelerometry data.

Due to varying accelerometer types, wear locations, and file types, we developed a process to derive minute- and day-level accelerometry summaries for each participant in each study at baseline and postintervention. First, we summarized axis counts for each individual at each data collection time point by 1-minute epochs and derived a wear-time indicator variable using a previously validated wear-time algorithm [60]. Next, an indicator variable was derived that categorized each minute as moderate-to-vigorous physical activity (MVPA) based on the axis count threshold that aligned with the accelerometer type, wear location, and age of the participant [61-66]. Because we did not consistently have triaxial data across all trials, we chose to use only the vertical axis counts to determine wear time and MVPA.

One trial used wrist-worn GENEActiv devices and provided the raw accelerometry data in gravitational units. For this study, wear time was determined using an SD-based approach developed by van Hees et al [67,68]. This approach uses a 60-minute time window with 15-minute overlapping increments. Physical activity thresholds for this study were based on the mean acceleration per epoch rather than counts per minute to be consistent with available validation studies for GENEActiv accelerometry data [69].

To establish MVPA thresholds used for examining physical activity, we conducted a literature review of MVPA threshold validation studies for all types of accelerometers across all participant ages represented in participating trials, as well as thresholds used by participating trialists. Using this information, we chose appropriate MVPA thresholds that (1) considered vertical axis counts only (as opposed to a triaxial vector magnitude measure), (2) were most frequently used by InSPACE trialists, and (3) were well established and commonly used in other physical activity studies (Table 5). Once a threshold was chosen for each accelerometer type, wear location, and age group combination represented the pooled dataset, we applied the chosen thresholds to the pooled accelerometry data to create harmonized PA measures across trials. In cases where age was missing for an individual, study population information was used to categorize the individual as adult, child, or older adult to choose an appropriate MVPA cut point. The current process has documented the accelerometer types, wear location, and age group combinations that are present across all currently recruited trials. If additional trials were to be recruited, the InSPACE investigator team would conduct a similar process for establishing the appropriate MVPA threshold, using existing validation studies that are relevant for the accelerometer type, wear location, and age group as well as the MVPA threshold used by the trial to inform the decision-making process. Raw accelerometry data for all studies will also be available to researchers to allow for different processing and harmonization approaches or to conduct sensitivity analyses.

**Table 5.** Moderate-to-vigorous physical activity thresholds by accelerometer type, wear location, and participant age.

Accelerometer	MVPA^a^ threshold
Actigraph	
Wrist	
Adults (18 - 65)	4730 cpm^b^
Hip	
Children (<18)	2296 cpm^c^
Adults (18-65)	1952 cpm^d^
Older adults (>65)	1041 cpm^e^
Actical	
Hip	
Adults (≥18)	1535 cpm^f^
Children (<18)	1600 cpm^f^
ActivPAL	
Thigh	
Adults (18-65)	12,028 cpm^g^
GeneActiv	
Wrist	
Adults (18-65)	93.2 mg^h^
Children (<18)	191.6 mg^h^

a MVPA: moderate-to-vigorous physical activity.

b Counts per minute; weighted average of calibrated thresholds [64,66].

c Calibrated on uniaxial counts on children 5‐8 years old [62].

d Calibration of uniaxial counts [63].

e Calibration of uniaxial counts [61].

f Calibration of uniaxial counts [70].

g Based on 3007 counts per 15-second epoch [65].

h Threshold based on average gravitational units (mg) [69,71].

**Figure 1.**
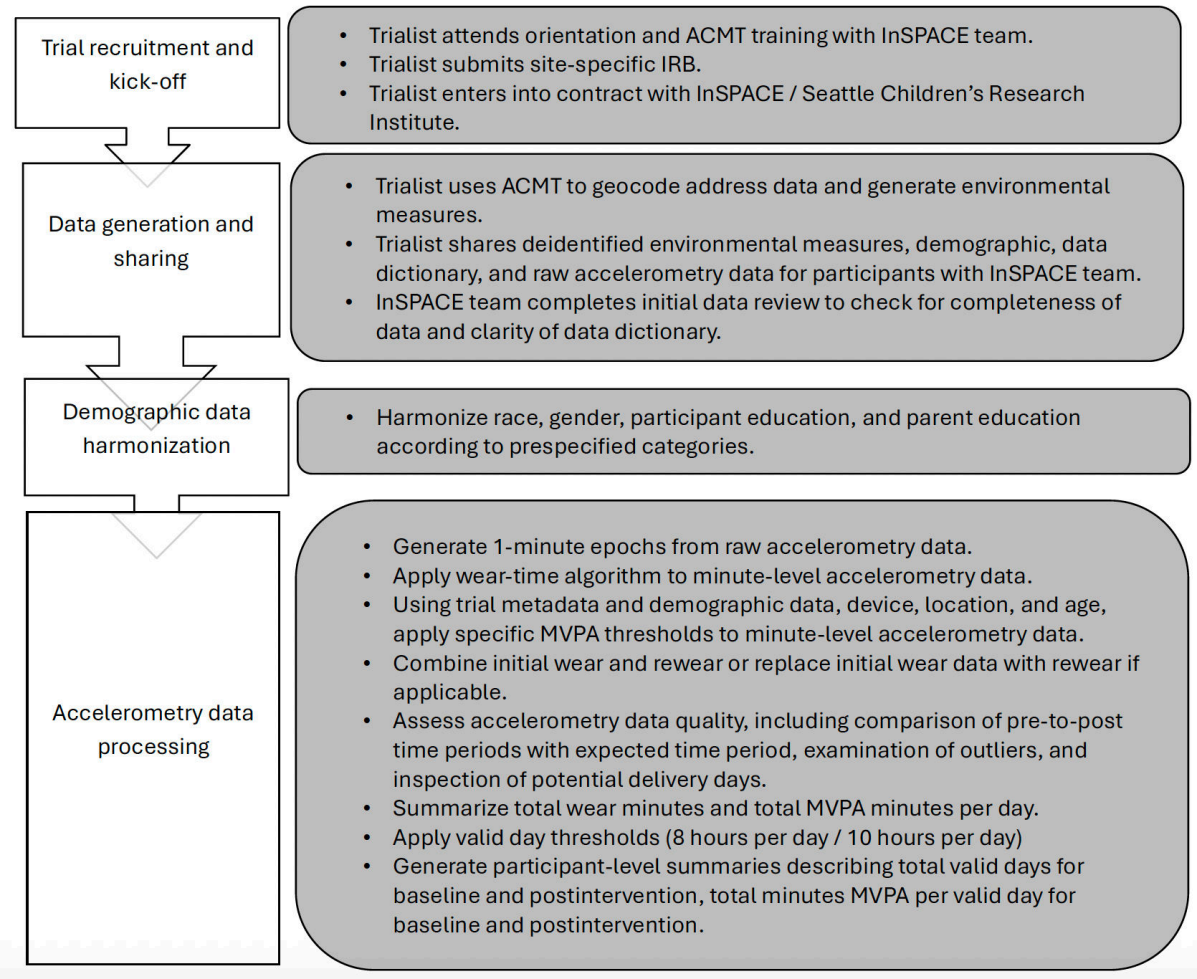
Interventions Supporting Physical Activity and the Environment data workflow diagram. ACMT: Automatic Context Measurement Tool; InSPACE: Interventions Supporting Physical Activity and the Environment; IRB: institutional review board; MVPA: moderate-to-vigorous physical activity.

Day-level summaries for each participant provided the total wear minutes per day, which were used to determine whether a given day was valid. Accelerometry days were considered valid if the day included 8 or more hours of wear time [59]. Of the included trials, 95% (n=37) trials requested that participants wear the accelerometer for 7 days at each assessment time point. One trial had participants wear accelerometers continuously throughout the intervention and into the postintervention period, so we extracted 7 days postintervention from the continuous data collection. Participants with fewer than 3 days of valid accelerometry data at baseline or postintervention were excluded. An exception was made for data from one trial that collected only 2 days of accelerometry data at each time point. For this trial, any participant with at least 2 valid days with 8 or more hours of wear time for baseline and postintervention was included. A final wide-format InSPACE accelerometry dataset was created that included a variable for the mean minutes of MVPA per valid baseline day, mean minutes of MVPA per valid postintervention day, the type of accelerometer and the specific threshold used to define MVPA, total valid days at baseline, and total valid days at postintervention. Figure 1 shows the full cycle of trial recruitment and data collection and processing.

#### Accelerometry Rewear Data

In some cases, trialists recontacted individuals whose initial accelerometry data did not provide adequate data (ie, not enough valid wear days) and asked participants to wear the accelerometer again. For these “rewear” scenarios, we applied the following logic: if valid rewear data were available and initial wear data did not have any valid days, only the rewear data were used. If both the initial wear data and rewear data contained valid wear days, we used the initial wear data up to the first valid rewear day and combined the initial wear data with all valid rewear days.

#### Visual Inspection of Delivery Days

Several quality assurance reports were generated to help us validate accelerometry data. We did not have complete data from trialists about when and if accelerometers were delivered or returned via mail. Some trials gave accelerometers to participants in person, and some mailed them, and at least 1 trial removed delivery days prior to sharing their accelerometry with the InSPACE team. While previous studies have used machine learning to develop algorithms for identifying delivery days (Moore et al [72]), the inconsistencies in expected delivery days and the lack of triaxial data across all InSPACE trials required us to use a combination of algorithm and manual review to identify potential delivery days.

**Figure 2.**
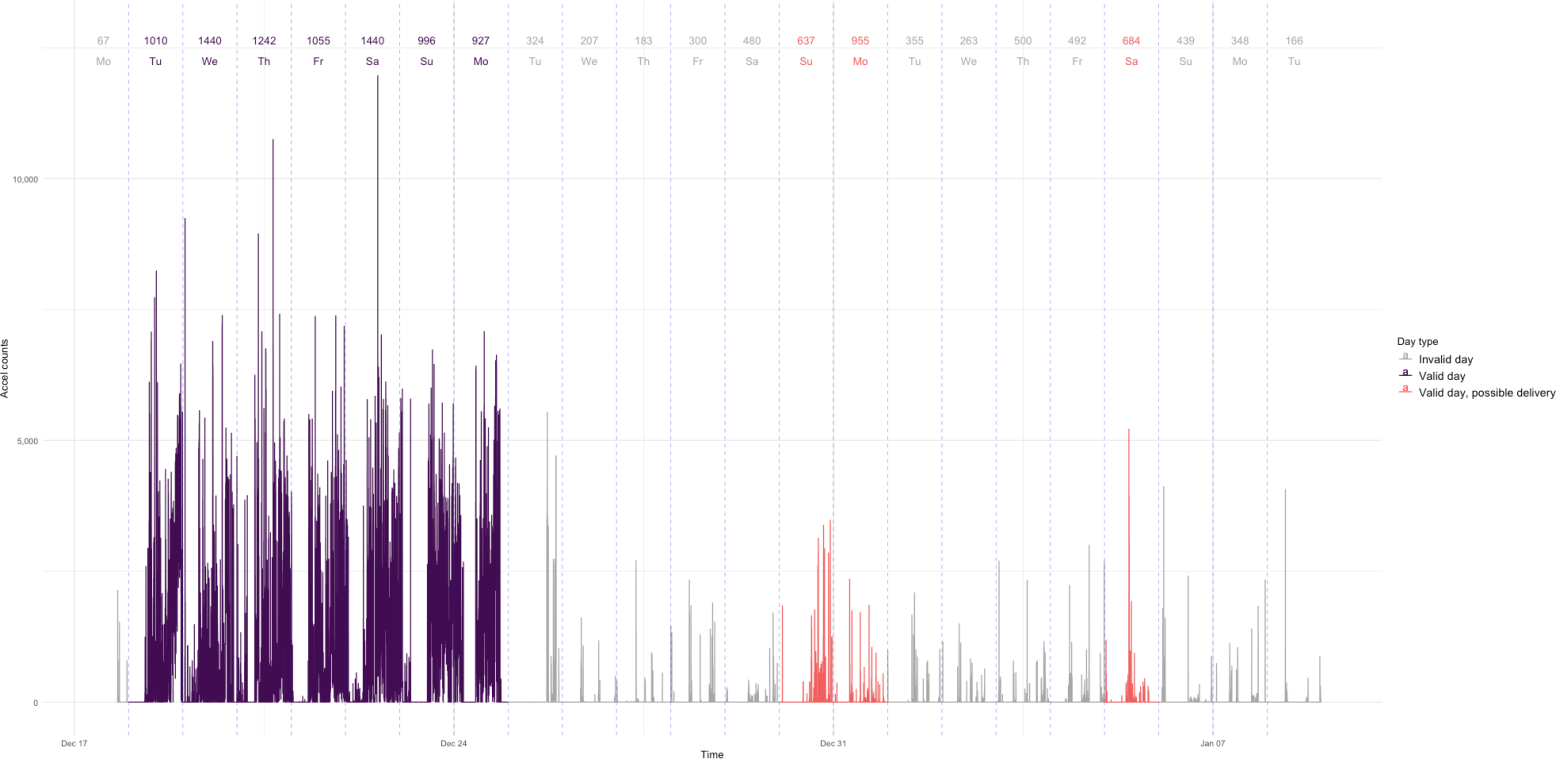
Plot of potential delivery days for an individual baseline data collection period with wear minutes per day at the top.

In reviewing the pooled accelerometry data, we were primarily concerned with days that were marked as a valid wear day (ie, 480 or more minutes of wear time) but were capturing movement of the accelerometer in transit. Days that were already marked as invalid due to insufficient wear time were not reviewed as potential delivery days, given that they were already excluded from analysis. In a manual review of data from 2 trials, we identified patterns that resembled potential delivery days. Participants were expected to wear devices for 7 consecutive days, so we identified deviations from this pattern, such as 1 or 2 days marked as a valid wear day, followed by or preceded by 2 or more invalid days (Figure 2), potentially indicating the device was in transit. When this pattern was identified for individuals who had 4 or more consecutive days of valid wear time, we flagged these individuals for manual review. Flagged days were visually inspected using a plot of accelerometer counts (refer to Figure 2 for a visual of marked possible delivery days) and confirmed as likely delivery (nonwear) days.

#### Accelerometer Data Collection Dates

Additional QA reports were created to examine the time elapsed between the baseline accelerometry data collection and postintervention data collection. Time between data collection periods was compared to expected time elapsed based on trial metadata (eg, the planned length of time of the intervention) to ensure that accelerometry data files were properly sorted into baseline and postintervention files, and that there were no accelerometry data that were potentially overlapping in time with the trial intervention period for the participant, particularly for interventions that included an intervention team-facilitated physical activity component. In cases where baseline and postintervention accelerometry data overlapped, accelerometry start dates were compared to expected start dates for accelerometry data collection for baseline and postintervention, and only one set of accelerometry data was retained, while the other was omitted due to overlapping data collection period. If no other accelerometry data were available for the individual for the missing data collection period, the individual was omitted from the analysis.

#### Ethical Considerations

The InSPACE project was approved by Seattle Children’s IRB (STUDY00002955). Because of the nature of InSPACE and the fact that the trials whose data were contributed to the data pool were already covered under the IRB of the trialist’s institution, this study was determined not to require a single-site IRB. The InSPACE study team provided boilerplate IRB language to study teams and offered individual consultation regarding approaches for seeking IRB approval at the trial site. These template documents were updated throughout the recruitment period in response to patterns and common questions that emerged from working with many teams over time. Given that all recruited trials chose to have their own trial team geocode and generate environmental measures, only deidentified data were shared with the InSPACE team, allowing for a much simpler IRB process at the pooled level.

IRB review and approval for participating trials generally followed one of four patterns: (1) Most commonly, the site IRB considered the InSPACE work exempt, and no modifications were required. (2) The trial had existing data sharing language in its protocol or consent documents and had to create a modification and get IRB approval for the new use of the data as a part of InSPACE. (3) The site did not have data sharing language already in their protocol or consent documents and had to create and get IRB approval for a modification requesting a waiver of reconsent and adding data sharing to the protocol. (4) The IRB did not approve the use of the participants’ home address data. This fourth scenario arose most often when the trial’s investigator had changed institutions and was not allowed to transfer identifiable data as part of the move. In some cases, trials were closed with their IRB and needed to be reopened before modification requests and approvals could be made.

## Results

As part of the InSPACE project, we recruited 39 physical activity intervention trials across the United States as of August 2025. Trial sample sizes ranged from 32 participants to over 500 participants. Nine of the trials explicitly recruited children (<18 years old), 2 of which recruited parent-child dyads. Trials with parent-child or 2-adult (couple-dyads) are identified by household or family IDs to account for their identical environmental exposures. Trial recruitment is completed as of August 2025, and analysis of the additional 8 recruited trials will be completed in 2026, with results expected to be published in late 2026.

One initially recruited trial was ultimately unable to locate accelerometry data and was thus omitted. Two participating trials discovered after entering into a contract with InSPACE that they only had accelerometry data available at the day level, summarized using trial-specific wear-time validation and MVPA thresholds. These data were retained in the InSPACE pool but flagged due to the potential lack of consistency in PA summarization. Additionally, we omitted participants from one trial whose postintervention accelerometry data were collected after March 2020, due to the potential influence of COVID-19 restrictions on physical activity.

All recruited trials collected data on age and either collected data on sex/gender or explicitly recruited only participants of a particular sex/gender. In 94% of the trials, race was either explicitly collected or recruitment was restricted to a particular racial group. The availability of additional demographic variables across the recruited trials is shown in Table 6.

Of the 39 recruited trials as of August 2025, we have completed data processing and harmonization for 31 trials. Some trials provided some data for all potential participants, including those who were not ultimately randomized, while others only included randomized participants. A total of 4471 participants who had been randomized to an intervention were included in the pooled data. Of those randomized, 97.5% (4360) of participants had addressed that InSPACE trialists were able to geocode and link to environmental measures, and 76.4% (3417) of randomized participants had at least 1 valid day of accelerometry data, with 49.4% (2208) of the randomized participants having complete, valid baseline and postintervention data.

**Table 6.** Availability of demographic variables across trials (N=31).

Demographic variable	Trials that collected data, n (%)
Age	31 (100)
Race^a^	29 (94)
Ethnicity^a^	29 (94)
Gender^a^	31 (100)
Education (adult trials)	22 (71)
Parent education (trials with children)	31 (100)
Income	24 (77)
Household size	15 (48)

a Includes trials that did not explicitly collect the demographic variable, but whose study population was selected based on a specific demographic characteristic.

## Discussion

### Data Pooling

To date, research on whether the built environment can modify the effectiveness of physical activity interventions has been limited by small sample sizes and the limited geographic spread of participants. The InSPACE project is creating a pooled dataset of physical activity intervention trial participants along with derived individual participant home neighborhood environmental attributes to be used in studying the role of the environment in influencing physical activity promotion. We have also documented our processes of data collection and harmonization for use in future work.

### Strengths and Challenges

There are numerous strengths of the pooled InSPACE dataset. We used standardized methods to analyze all accelerometry data to ensure consistency in defining wear time and operationalizing MVPA. We also linked standardized environmental measures using our own standardized interpolation methods to ensure consistency across all pooled trials, rather than harmonizing any trial-specific measures. Trialists themselves used the ACMT to geocode and generate environmental measures, so no sharing of address or other identifying data was required, greatly streamlining the IRB process for the InSPACE team and for participating trialists. We included numerous environmental measures of interest, including attributes of the social, economic, and built environment allowing us to explore several potential pathways through which the environment may affect physical activity changes within intervention trials.

The InSPACE study is further strengthened by the input of numerous experts in the field of physical intervention trials who made up the SAC. These individuals assisted us in identifying and recruiting trials across the country to help ensure the diversity of participants in the pooled trial and a variety of intervention types.

We also faced several challenges both in recruiting trials and in harmonizing trial data. Due to the challenges of harmonizing income data across trials and the considerably different income categories captured across trials, we do not have a consistent measure of income or socioeconomic status for participants in the pooled data but rather maintain the study-specific income variables. This approach reduces potential bias that may be introduced in harmonizing vastly different income groups but also makes comparisons by individual income levels more complex. Despite these limitations in harmonizing income, we do have harmonized education level for participants themselves (for adults) or for the parent of the participant (for children) in 94% of the pooled trials, which may provide a proxy for socioeconomic status.

Additionally, while we created standard measures of MVPA across trials using existing validation studies for the specific accelerometry type and wear location, there is potential for error in using standardized uniaxial count-based measures of physical activity, and there remains a lack of consensus on the accurate count threshold for hip-worn accelerometers among older adults and for wrist-worn devices among all ages. Different thresholds or approaches to measuring MVPA with accelerometry data can result in vastly different measures of physical activity [73,74]. However, uniaxial count-based measures are a convenient and well-studied method of measuring physical activity that can be applied to numerous types of devices, including both uniaxial and triaxial.

### Next Steps

Since we anticipate that the InSPACE data pool might be usable to address questions outside of the primary aims of InSPACE, we developed a process for the use of pooled data outside of the scope of InSPACE. Language regarding this process was included in the data use agreement with consortium trials. Proposals from the InSPACE consortium trialists or from anyone outside the InSPACE Investigator team are first reviewed by the InSPACE Investigator team where they determine whether the proposed research question (1) provides an important contribution to the field and is addressable using InSPACE data, (2) is outside of the original InSPACE aims, and (3) is within the scope of InSPACE (ie, focuses on environmental factors related to physical activity). Once approval from the InSPACE investigators’ team is obtained, the proposal is brought to the SAC for final approval. For proposals that fall outside the scope of InSPACE, additional consent from all participating trialists is required for access to their data. Proposals brought from the InSPACE Investigators team do not require SAC approval if the proposed research question falls within the scope of the InSPACE. We are hopeful that the InSPACE data pool will provide opportunities to address additional interesting questions around physical activity and neighborhood environment.

### Conclusion

Despite the challenges in gathering and harmonizing data across InSPACE trials, the resulting pooled dataset provides a unique opportunity to examine the potential effect of the environment on physical activity changes in the context of interventions seeking to increase this behavior.

## Abbreviations

ACMT: Automatic Context Measurement Tool
ACS: American Community Survey
CDC: Centers for Disease Control and Prevention
InSPACE: Interventions Supporting Physical Activity and the Environment
IRB: institutional review board
MVPA: moderate-to-vigorous physical activity
NIH: National Institutes of Health
NLCD: National Land Cover Database
PA: physical activity
SAC: scientific advisory council
